# Early epidemiological characteristics explain the chance of population-level virus persistence following spillover events

**DOI:** 10.1371/journal.pbio.3003315

**Published:** 2025-08-21

**Authors:** Clara L. Shaw, David A. Kennedy

**Affiliations:** 1 Department of Biology and the Huck Institutes of the Life Sciences, The Pennsylvania State University, University Park, Pennsylvania, United States of America; 2 Department of Biology, University of Minnesota Duluth, Duluth, Minnesota, United States of America; Northern Arizona University, UNITED STATES OF AMERICA

## Abstract

Spillover of viruses into novel host species occurs frequently. Often, spillover results in dead-end infections in novel hosts, sometimes, in stuttering transmission chains that die out, and rarely, in large epidemics with sustained transmission. If we could identify early which outcome will occur following a spillover event, we could more appropriately invest in efforts to surveil, respond to, or prevent disease emergence. Our goal was to identify early epidemiological characteristics that correlate with these outcomes, including those predictive of population-level virus persistence in novel hosts. To identify these characteristics, we experimentally induced spillover in the *Caenorhabditis* nematode-Orsay virus system and measured infection prevalence in exposed populations and virus shedding and infection intensity from infected hosts in replicate populations of eight strains belonging to seven non-native host species. We then passaged 20 adult nematodes from exposed populations to virus-free plates where they reproduced, initiating new populations to which they had the potential to transmit virus. We used quantitative PCR to track virus presence in passaged host populations for 10 passages or until virus was undetectable, indicating its loss. We then used a correlative modeling and a mechanistic modeling approach to understand which epidemiological characteristics were associated with population-level viral persistence. In our correlative models, we found that the number of passages until virus loss was associated with early epidemiological characteristics in the spillover host populations, including infection prevalence in the initially exposed population, the ability of hosts to detectably shed the virus, and the relative susceptibility of the host species, but not infection intensity. When all these characteristics were included simultaneously in a correlative model, only infection prevalence and shedding were significantly associated with virus maintenance, and the model explained over half of the variation in the data. We then developed a mechanistic model that attempts to explain virus passage success by using our epidemiological characteristics data to calculate the probability that at least one worm infectious enough to infect a conspecific is transferred during passage. This mechanistic model explained 38% of the variation in the data on its own. With the goal of understanding how our mechanistic model falls short, we used model selection to test a suite of larger models that included or excluded each epidemiological characteristic and included random effects of strain, experimental line, passage number, and block while the mechanistic prediction was included as an offset. We found that 66% of the variation in our data could be explained by a model that included our mechanistic prediction in addition to infection prevalence, infection intensity, and random effects. Altogether, our study demonstrates that early epidemiological characteristics can play a substantial role in explaining the ultimate outcome of a spillover event.

## Abstract 2

Virus spillover into new host species can lead to outcomes ranging from dead-end infections to sustained epidemics. This study uses a nematode-virus model to show that early spillover traits, especially infection prevalence and shedding, are important predictors of viral persistence.

## Introduction

Pathogens are continuously exchanged between species [1–4], yet pandemics following cross-species transmission are relatively rare [5–7]. This demonstrates that most spillovers are not a threat beyond the infected individual and, perhaps, a handful of nearby contacts. However, the rare event where a pathogen spills over and sustainably transmits in the recipient population can have catastrophic consequences (e.g., HIV [8], SARS-CoV-2 [9], *Batrachochytrium dendrobatidis* [10], white-nose syndrome [11], and potato blight [12]). With many spillovers and few emergence events, a major challenge of today’s research on infectious disease is to predict which pathogens have the potential to emerge in new populations.

Efforts to anticipate disease emergence events have included approaches such as correlating characteristics of the reservoir host, virus, and ecological context with previous spillover (and in some cases, emergence) events [13–16] and machine learning to extract viral genomic signatures of zoonotic potential [17,18]. Several risk factors for spillover to humans have been identified with these methods including that viruses are more likely to spill over if they infect reservoir hosts closely related to humans [13,16], if they can replicate in the cell cytoplasm instead of the nucleus [13], if they establish long-term infections [14], if they infect reservoir hosts whose ranges overlap high density human populations [13,15,16], and if genomes contain features of human-infecting viruses [17]. Nevertheless, the predictive value of these factors is limited because these elevated-risk situations are common, despite instances of viral emergence being very rare [5,6]. Furthermore, even for viruses that have emerged in the past (e.g., Ebola), there have also been many spillover events in which the virus failed to emerge [19]. This suggests that the characteristics of viruses and hosts alone are not sufficient for predicting the risk of emergence after a given spillover event but rather that each spillover event has a unique chance of resulting in emergence.

Rather than attempts at prediction based on the above characteristics, some have advocated for increased surveillance to identify events where a virus spreads following spillover [6]. The underlying assumption of this approach is that transmission plays a large role in determining the likelihood of emergence. We extend this logic by arguing that it may be possible to link the early epidemiological characteristics following a spillover event to the probability of emergence in the novel host population. If correct, measuring these epidemiological characteristics could aid efforts to appropriately invest in responses to future spillover events and direct resources toward those that pose the greatest risk for emergence.

To persist in a novel host population following spillover, a virus must overcome barriers to replication within spillover hosts and barriers to transmission between them [7,20,21]. Within spillover hosts, the virus must evade the immune system, enter cells, replicate, assemble, and be shed (i.e., emerge from the body of the primarily infected individual) [21]. To transmit among spillover hosts, the virus must encounter these host individuals at doses required to infect them [22]. Population-level persistence in the spillover host will fail to occur if any of these processes are inhibited, or if they do not occur reliably enough to overcome stochastic breaks in this chain. If we could quantify the probability of these steps succeeding, we could mechanistically understand and quantify the likelihood of emergence after a spillover event.

Here we use a model system, multiple species of *Caenorhabditis* nematodes and Orsay virus, a natural virus of *Caenorhabditis elegans* [23,24], to study the link between the early epidemiological characteristics of an outbreak following spillover into a novel host population (spillover outbreak) and the outcome regarding population-level persistence (hereafter referred to as “maintenance”) of the virus in that spillover host population (i.e., immediate loss, loss after minimal transmission, or long-term maintenance). We had the additional goal of improving our understanding of the emergence process by mechanistically linking virus maintenance to the epidemiological characteristics of a spillover event.

Orsay virus is a bipartite, positive sense RNA virus that naturally infects the gut cells of *C. elegans* nematodes and is spread by the fecal-oral route [25]. Virulence in immune-competent *C. elegans* is generally mild, with a brief delay in reproduction associated with virus infection [24]. Various defenses in *C. elegans* modulate virulence and virus amplification within infected populations [26], and worms tend to recover from infection after a few days [27]. Our previous work has shown that certain *Caenorhabditis* species in addition to *C. elegans* can become infected by Orsay virus, but these species vary in their ability to transmit it [23].

**Fig 1:**
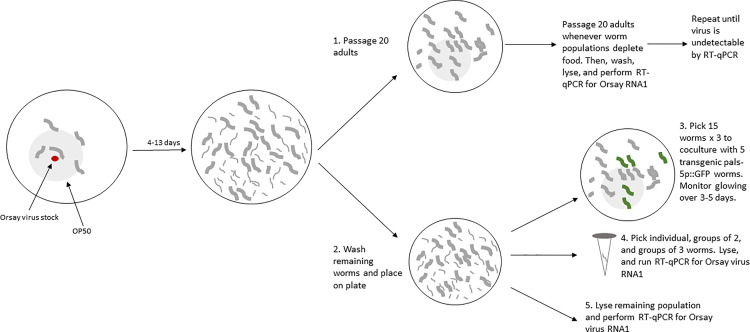
Experimental design depicting the passage experiment and the methods for quantifying infection prevalence, shedding ability, and infection prevalence in spillover populations. Relative susceptibility of host strains was determined in a separate experiment (see methods). We initiated spillovers across eight novel host strains and passaged 20 adult animals once bacterial food (OP50) was depleted (4–13 days) to maintain host populations, and potentially, virus transmission (1). Passages were repeated whenever food was depleted. Non-passaged animals remaining on the plate were lysed and assessed for viral RNA1 with RT-qPCR. The non-passaged worms from the initial spillover populations were washed and again placed on plates (2). Animals were picked either to quantify viral shedding (3) or infection prevalence and intensity (4). Viral shedding was assessed by coculturing groups of 15 worms from the spillover plate with five highly susceptible transgenic fluorescent reporter animals (pals-5p::GFP *C. elegans* strain *jyIs8;rde-1* [28]); plates were monitored for 3–5 days for glowing. Infection prevalence and infection intensity were assessed by RT-qPCR for Orsay virus RNA1 on groups of 1–3 lysed worms. Finally, we performed RT-qPCR for Orsay virus RNA1 using the remaining worms from the exposure population (5).

In our experiment, we induced spillovers across eight nematode strains belonging to seven *Caenorhabditis* species that were previously determined to be at least partially susceptible to Orsay virus [23]. We quantified the prevalence of infection, viral shedding, and infection intensity (viral genomes within infected animals) and repeatedly passaged subsets of populations to new plates to assay viral maintenance over time (Fig 1). We show that viral maintenance is positively correlated with infection prevalence in exposed populations, viral shedding in these populations, and susceptibility of hosts, but not with the intensity of infection in infected spillover hosts. We then constructed a mechanistic model of virus transmission and tested its value in a suite of statistical models, which demonstrated that some variation in virus maintenance can be explained by our current understanding of disease dynamics (the mechanistic component of the model), but there were extra effects of several factors including infection prevalence, infection intensity, and the host strain.

## Results

### Epidemiological characteristics in spillover populations are correlated with virus maintenance

**Fig 2:**
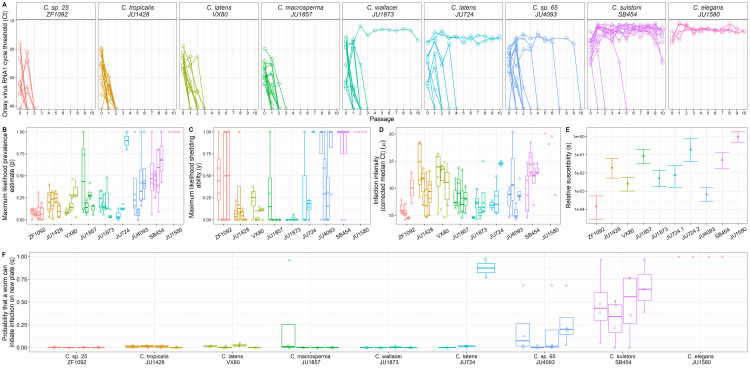
Virus was maintained variably across *Caenorhabditis* species/strains, which also differed in their epidemiological characteristics of spillover and chance of virus maintenance predicted from our mechanistic model. **A)** Host strains are ordered by the number of virus-positive plates over the course of the experiment (positive control *C. elegans* strain JU1580 set farthest right). Passage 0 denotes the exposure population. Note that the extraction procedure for passage 0 plates was different from the subsequent passages; many adult worms were removed from passage 0 populations to measure infection prevalence, intensity, and shedding (see methods). Strains differed in epidemiological characteristics including **B)** maximum likelihood prevalence of infection, **C)** infection intensity (corrected median Ct), **D)** maximum likelihood shedding ability (the probability that 1 infected worm could cause visible fluorescence on a shedding plate), and **E)** relative susceptibility (TCID50/μL of the stock virus measured in each strain/thaw line divided by the TCID50/μL measured in *C. elegans* JU1580). **F)** Probability q that any single transferred worm is infected and shedding sufficiently well to initiate infection on a new plate, calculated for each experimental line from epidemiological characteristics data (infection prevalence on the exposure plate, shedding ability, and the probability that worms shed enough to infect a given strain). Experimental blocks are designated by shape and separate boxes in boxplots. Data for the positive control JU1580 plates (single line per block) are shown but were not included in statistical models since infection of this highly susceptible *C. elegans* strain is not “spillover.” The data underlying this figure can be found in https://doi.org/10.5281/zenodo.15739577.

To identify epidemiological characteristics that correlate with virus maintenance in spillover populations, we exposed eight strains of *Caenorhabditis* to Orsay virus, a natural virus of *C. elegans* [24], to initiate spillover outbreaks. For each host strain, we generated 16 replicate virus lineages spread across four independent experiments (hereafter, “blocks”). The *Caenorhabditis* strains used here have been previously determined to be susceptible to Orsay virus and variable in their ability to maintain the virus through passaging [23]. Across the experiment, 15 experimental lines displayed virus maintenance with detectable virus (by RT-qPCR for Orsay virus RNA1, Ct < 40) through 10 passage events where 20 randomly selected individuals were transferred to new plates. Of the lines that showed virus maintenance, 12 were in the *Caenorhabditis sulstoni* strain SB454, two were in the *Caenorhabditis latens* strain JU724 (second thaw (JU724.2)), and one was in the *Caenorhabditis wallacei* strain JU1873 (Fig 2A). Virus was detectable across all 10 passages for positive control lines of *C. elegans* JU1580 in the first three blocks (Fig 2A). The positive control in the last block became contaminated with non-OP50 bacteria and was discarded after the eighth passage plate despite virus still being detectable (Fig 2A).

We quantified epidemiological characteristics of spillover by measuring infection prevalence, infection intensity, and shedding in populations exposed to the original virus inoculum (“spillover populations,” Fig 1). To measure infection prevalence, three individuals, three groups of two, and nine groups of three worms were collected at random from spillover populations and lysed. This sampling technique was used to efficiently quantify infection prevalence regardless of whether prevalence was high or low. RT-qPCR for Orsay virus RNA1 was performed on lysates to determine infection prevalence (fraction of virus-positive worms) and infection intensity (median Ct of virus-positive worms). We determined the experimental worms’ ability to shed the virus by coculturing worms transferred from experimental plates with the transgenic fluorescent pals-5p::GFP *C. elegans* strain *jyIs8;rde-1* [28] and assessing fluorescence on these plates for 3−5 days. The presence of fluorescing worms during this period indicated that experimental worms transmitted the virus to the sentinel *jyIs8;rde-1* animals. These “shedding plates” were initiated with 15 adult experimental animals and five L4-adult *jyIs8;rde-1* individuals. We used three shedding plates per experimental line when a sufficient number of worms were available (113 out of 118 assays). Maximum likelihood estimates of prevalence and shedding ability were calculated by maximizing the likelihood of prevalence and shedding results across both assays (see methods). Finally, although susceptibility had previously been documented [23], we more precisely measured the relative susceptibility of each strain in a separate experiment by modulating dose to determine the median tissue culture infectious dose (TCID50) per μL of the virus stock in each worm strain [29] and dividing this by the TCID50/μL of the same batch of virus in the highly susceptible positive control strain *C. elegans* JU1580.

We used (generalized) linear models to explain variation in prevalence, median infection intensity, and shedding ability due to strain and block, which were treated as fixed effects in these models [30]. Differences in prevalence and shedding ability were modeled using the quasibinomial family and differences in median infection intensity were modeled with the Gaussian family. Strains differed in prevalence of infection in exposed populations (strain: F = 6.99, *p* < 0.001; Fig 2B), median intensity of infection in infected exposed hosts (strain: F = 5.33, *p* < 0.001; Fig 2C), and their ability to shed the virus (strain: F = 10.68, *p* < 0.001; Fig 2D). For shedding ability, there was also a significant effect of block (F = 2.99, *P* = 0.03). We used likelihood-based inference to determine that strains also differed in relative susceptibilities (χ^2^ = 324.4, df = 9, *P* < 0.0001) and ranged from 0.0001X to 0.1922X relative to highly susceptible *C. elegans* strain JU1580 (Fig 2E).

**Fig 3:**
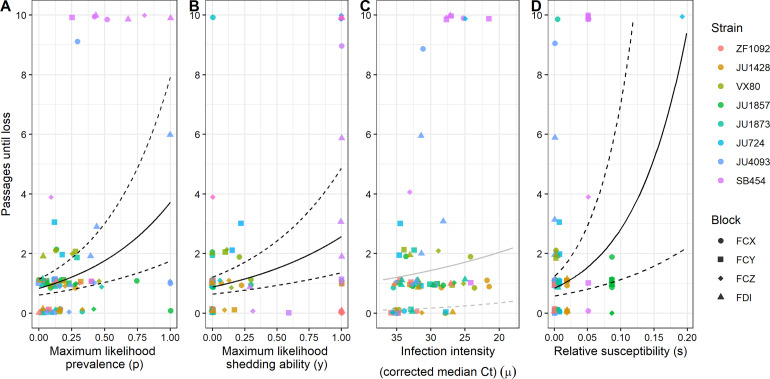
The duration of virus maintenance is associated with A) infection prevalence in exposed populations, B) the ability of exposed hosts to shed the virus, and D) the relative susceptibility of strains, but not C) the intensity of infection in infected hosts. Points are slightly jittered along the *y* axis for visibility. Solid black lines indicate significant relationships from single-factor models. Solid gray lines indicate non-significant relationships. Dashed lines indicate ± 1 standard error. Associations between virus maintenance and epidemiological characteristics by strain are shown in S1, S2, and S3 Figs. Correlations among epidemiological characteristics are shown in S4 Fig. The data underlying this figure can be found in https://doi.org/10.5281/zenodo.15739577.

To identify the epidemiological characteristics that associate with the duration of virus maintenance through passaging, we ran generalized linear mixed-effects models with negative binomial error. We ran one model for each individual factor (where all models also included random effects of strain and block), which showed that the duration of virus maintenance was positively correlated with the prevalence of infection (estimate = 1.49 ± st. error 0.44, χ^2^ = 11.56, *p* < 0.001, marginal R^2^ = 0.17, Fig 3A), shedding ability (estimate = 1.07 ± 0.32, χ^2^ = 10.72, *p* = 0.001, marginal R^2^ = 0.23, Fig 3B), and relative susceptibility (estimate = 12.12 ± 5.42, χ^2^ = 4.27, *p* = 0.039, marginal R^2^ = 0.12, Fig 3D) but not with the median infection intensity (Ct value) of infected worms (estimate = −0.04 ± 0.03, χ^2^ = 1.06, *p* = 0.303, marginal R^2^ = 0.01, Fig 3C). We next ran another similar generalized linear model with negative binomial error except that all epidemiological characteristics were included as fixed effects simultaneously. This model found that the duration of virus maintenance was positively associated with infection prevalence (estimate = 1.11 ± 0.43, χ^2^ = 6.68, *p* = 0.010) and shedding ability (estimate = 1.57 ± 0.34, χ^2^ = 17.53, *p* < 0.001), but not median infection intensity of infected animals (estimate = 0.01 ± 0.03, χ^2^ = 0.15, *p* = 0.70) or relative susceptibility (estimate = 2.32 ± 2.96, χ^2^ = 0.62, *p* = 0.43). Overall, the combined fixed effects in this model had a marginal R^2^ of 0.52, meaning that the majority of variation in the duration of virus maintenance in our experiment can be explained by the early epidemiological characteristics of the spillover outbreak.

### Mechanistic model of maintenance explains variation in the data

To better explore the potential for a causative relationship between our epidemiological characteristics and virus maintenance, we developed and tested a mechanistic model that uses our epidemiological characteristics data to explain passage success. Notably, the parameters in this model are not fit to our data on passage success, but rather, predictions about passage success are generated by feeding the epidemiological characteristics into the model. Our mechanistic model arises from the binomial distribution and has the basic form:


$$Prob(Passage\;success)=1-(1-{q)}^{n}\mathrm{\backslash ]}$$ (1)

where q is the probability that any single transferred worm is infected and shedding sufficiently well to initiate infection on a new plate with conspecifics, and n is the number of worms transferred, which was 20 in our experiment. q depends on infection prevalence on the exposure plate p, shedding ability given infection y, and the probability that worms shed enough to infect a given strain z (see methods). Essentially, the model asks, what is the probability of transferring at least one worm that can initiate an infection? To avoid passage success probability estimates of 0% or 100%, which could arise erroneously due to finite sample sizes in our assays for infection prevalence and shedding ability, we weighted our estimates toward intermediate values (see methods).

**Fig 4:**
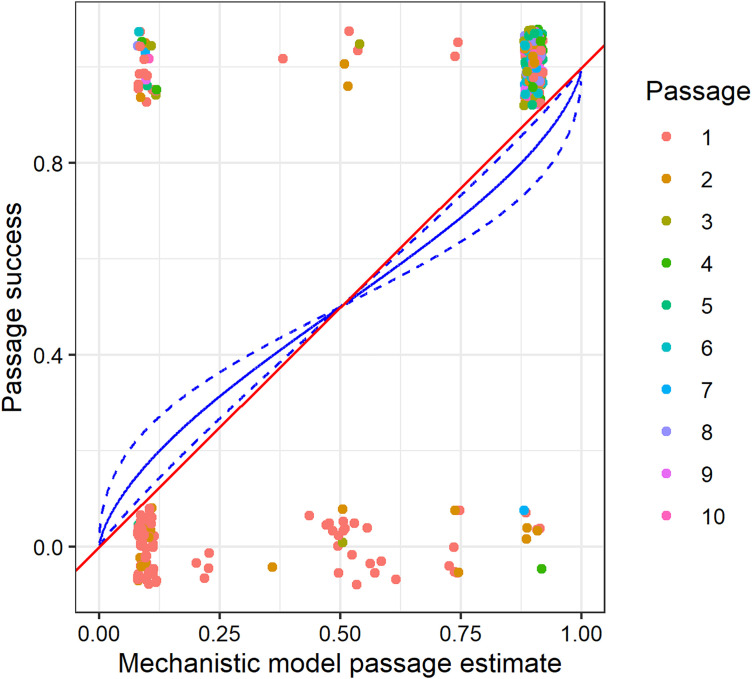
The mechanistic model predicted passage success correlates with actual passage success in the experiment. Points are jittered along the *y* axis for visibility. A 1-to-1 line is shown in red, and the mechanistic model prediction from a binomial generalized linear mixed-effects model with intercept = 0 and random effects of passage, experimental line, strain, and block is shown in blue. Dashed blue lines indicate ± 1 standard error. The data underlying this figure can be found in https://doi.org/10.5281/zenodo.15739577.

To determine how good our mechanistic model was at explaining the probability of successful viral passage (defined here as passage plates with viral Ct < 25), we created a binomial generalized linear model with no predictors other than the logit transformed probability of successful transfer as determined from equation 1. We forced the intercept through 0 since we expected a 1–1 relationship between our mechanistic model prediction and true passage success. The R^2^ of this model was 0.38, indicating that our mechanistic model can explain more than one third of the variation in virus maintenance across passages. We then ran a second version of this model that allowed for random effects of strain, experimental line, passage number, and block. This model had an overall R^2^ of 0.66, and the marginal R^2^ of the mechanistic effect alone was 0.20 (Fig 4). Interestingly, the improvement in variation explained by the second version is mostly due to the random effect of strain (marginal R^2^ = 0.36), suggesting that there is an effect of strain that is only partially captured by the mechanistic model. In addition, the random effect of experimental line accounted for a marginal R^2^ of 0.06, passage number accounted for a marginal R^2^ of 0.05, and block accounted for a marginal R^2^ of <0.01.

Next, we wanted to determine if any of our measured epidemiological characteristics had effects on virus maintenance beyond what we captured in our mechanistic model. To do so, we used the logit transformation of Equation 1 as an offset in a suite of 16 binomial generalized linear models that included or excluded each of the epidemiological characteristics. All models also included the random effects of strain, experimental line, passage number, and block. Models were compared by corrected Aikake Information Criteria (AICc) (Table 1; sample size = 289). The best model included infection prevalence and median infection intensity, but not shedding ability or relative susceptibility. In this best model, the effect of infection prevalence was positive, and the effect of median infection intensity was negative (which, because it is measured on a Ct scale, means that more virus within novel hosts translates to better passage success than predicted by the mechanistic effect alone). Analysis of the suite of models (AICc weights and model-averaged parameter estimates) also suggested that prevalence is important to virus maintenance beyond its role in the mechanistic model, and the 95% confidence intervals of the model-averaged parameter estimate did not overlap zero, indicating that prevalence had a positive effect on passage success above that explained by the mechanistic effect (Table 2). Likewise, median infection intensity had a high AICc weight, suggesting that it is an important component of the best models predicting passage success, but the model averaged parameter estimate overlapped 0 (Table 2). Thus, in comparison to the baseline set by the mechanistic effect, infection intensity is correlated with increased passage success in some models and decreased passage success in others.

**Table 1:** Model comparison of the suite of models including or excluding the four epidemiological characteristics of spillover in addition to an offset of the mechanistic model (Equation 1). All models also included the random effects of strain, experimental line, passage number, and block. The models used the binomial family. Marginal and conditional observation-level variance explained (R^2^m/R^2^c) was calculated using R package MuMIn version 1.48.4 [31]. Note that all models with reasonable support included effects of prevalence and infection intensity.

Model	AICc	Δ AICc	Akaike weight	R^2^m/R^2^c
Success ~ 0 + prevalence + intensity	158.3	0	0.434	0.25/0.66
Success ~ 0 + shedding + prevalence + intensity	159.3	1.0	0.251	0.21/0.62
Success ~ 0 + prevalence + intensity + rel. susc.	159.9	1.6	0.189	0.32/0.69
Success ~ 0 + shedding + prevalence + intensity + rel. susc.	160.9	2.6	0.116	0.27/0.65
Success ~ 0 + intensity + rel. susc.	167.3	9.0	0.005	0.16/0.57
Success ~ 0 + intensity	168.2	9.9	0.003	0.00/0.52
Success ~ 0 + shedding + intensity + rel. susc.	169.3	11.0	0.002	0.16/0.57
Success ~ 0 + shedding + intensity	169.9	11.6	0.001	0.01/0.53
Success ~ 0 + shedding + prevalence	185.4	27.1	0.000	0.05/0.57
Success ~ 0 + shedding + prevalence + rel. susc.	185.9	27.6	0.000	0.13/0.58
Success ~ 0 + prevalence	186.7	28.4	0.000	0.03/0.58
Success ~ 0 + rel. susc.	187.2	28.9	0.000	0.09/0.56
Success ~ 0 + shedding + rel. susc.	187.2	28.9	0.000	0.13/0.57
Success ~ 0	187.3	29.0	0.000	0.00/0.56
Success ~ 0 + prevalence + rel. susc.	188.0	29.7	0.000	0.08/0.58
Success ~ 0 + shedding	188.6	30.3	0.000	0.01/0.57

**Table 2:** Akaike weight of all models containing a given epidemiological characteristic and model-averaged parameter estimates for epidemiological characteristics from the suite of 16 binomial (logit link) generalized linear mixed-effects models where epidemiological characteristics were included or excluded but keeping the mechanistic effect (Equation 1) as an offset in all models. The random effects of strain, experimental line, passage number, and block were also included in all models.

Spillover characteristic	Akaike weight	Model-averaged parameter estimate on logit scale (95% conf. int)
Prevalence	0.989	5.27 (1.64, 8.89)
Shedding ability	0.370	−1.17 (−3.34, 1.00)
Rel. susceptibility	0.312	12.59 (−24.87, 50.05)
Infection intensity	1.000	−0.05 (−0.11, 0.01)

## Discussion

Predicting whether a spillover outbreak will lead to disease emergence in a new host species remains a challenge. Spillover and transmission are difficult to document and track in the field, and few experimental studies with replicate spillovers and natural virus transmission have been completed in animal hosts. In the studies that have been able to successfully accomplish this feat [32–34], disease emergence is almost never observed. In this study, we measured epidemiological characteristics of spillover outbreaks of Orsay virus in *Caenorhabditis* nematodes to discover which characteristics, if any, are predictive of virus maintenance after spillover. We first used a correlative approach and found that when each of our four epidemiological characteristics were analyzed separately, the prevalence of infection in spillover populations, the shedding ability of spillover hosts given infection, and the relative susceptibility of spillover host species were all positively correlated with virus maintenance in the spillover host species, suggesting that any of these three factors could potentially be useful early metrics to policy makers when determining how to respond to spillover outbreaks. In contrast, the median infection intensity of infected spillover host individuals was not a useful predictor of viral maintenance. When analyzing the data through multiple regression that simultaneously included all four epidemiological characteristics, we found that only infection prevalence and viral shedding ability were significant predictors but that these two factors could explain more than 50% of the variation in viral maintenance in the spillover host species. Next, with the goal of gaining a mechanistic understanding of virus maintenance in spillover hosts, we built and tested a mechanistic model that used the same epidemiological characteristics to predict passage success of the virus. Our mechanistic model explained 38% of the variation in virus passage success, but we found that additional variation was explained by host strain and that the epidemiological characteristics of infection prevalence and infection intensity are important determinants of virus passage success beyond their role in our mechanistic model.

### Current understanding of virus emergence is useful but incomplete

For virus emergence after spillover to occur, shedding from the spillover host must follow an initial successful infection. Then, the virus must contact and be able to exploit a new individual in the population [7,35]. We built our mechanistic model with these steps in mind to help us clarify how epidemiological characteristics combine to promote virus emergence. The success of our model at explaining virus passage data underscores that population-level viral persistence in spillover host populations requires the convergence of multiple epidemiological factors. For example, this can be visualized in Fig 1 where the two strains ZF1092 and JU1428 lose the virus extremely quickly, as predicted by the mechanistic model, even though ZF1092 has moderate to high shedding ability and JU1428 has moderate to high infection intensity and relative susceptibility. In these examples, the mechanistic model can anticipate very little passage success for both strains, when models built on individual epidemiological characteristics would be unable to explain both cases. We also saw cases where each of the epidemiological characteristics seemed to prevent maintenance despite moderate values for other characteristics. For example, passages died out when shedding ability was low even when infection prevalence was high (e.g., JU1857, JU1873), and when shedding ability was high but prevalence was low (e.g., ZF1092). In addition, moderate infection prevalence and shedding ability were still inadequate for virus maintenance when hosts were not very susceptible (e.g., JU4093). These results demonstrate that measuring a single epidemiological characteristic (although positively correlated with emergence) may be misleading regarding whether a spillover event is likely to result in disease emergence.

Our comparison of experimental data and theoretical predictions identified where our mechanistic model fell short. The incorporation of strain, line, passage number, and block as random effects improved model fit, and the epidemiological characteristics of infection prevalence and median infection intensity were important beyond their roles in the mechanistic model. It is quite likely that the strain and experimental line effects could be explained by host attributes that were not fully captured by the measured epidemiological characteristics (e.g., fecundity, growth rates, feeding rates, genetic variation) or additional characteristics of infection (e.g., infection duration/recovery rate, virulence). In addition, our model assumes that the epidemiological characteristics we measured in the initial population remained unchanged as the virus is passaged to subsequent populations, which is almost certainly incorrect and may be partially responsible for the imperfect fit of our mechanistic model to the data. Future work to determine the factors that underlie this remaining strain and experimental line level effect may help us further explain virus maintenance after spillover.

### Epidemiological characteristics of spillover vary in importance for predicting virus maintenance

Infection prevalence in spillover populations was the strongest predictor of future virus maintenance. While we note that our experimental setup does not distinguish between infection caused by the inoculum and infection caused by transmission between worms in the spillover population, this is how spillover is frequently observed in natural events (e.g., in some outbreaks of Nipah virus [36] or Ebola virus [37]) with clusters of cases that could have been transmitted directly from a reservoir or from human index cases. The importance of infection prevalence for virus maintenance is analogous to findings in the invasive species field where it has been shown that high propagule pressure increases the likelihood of establishment of invasive species in new habitats [38]. In the invasive species literature, this effect has been credited to the fact that increasing propagule pressure reduces the effects of demographic and environmental stochasticity and widens genetic bottlenecks [39]. Through our model comparison approach, we have identified a role of infection prevalence beyond its role in our mechanistic model. The extra benefit of high infection prevalence might be indicative of Allee effects during pathogen establishment, which might arise if there are dose–response effects such that two infectious worms are more than twice as infectious as one [40], which has been documented in some systems [41,42]. One could imagine other ways that high prevalence could be beneficial such as increasing rates of coinfection in cells that could lead to virus reassortment, potentially promoting adaptation to new hosts or reducing negative impacts of Mueller’s ratchet [43].

We had hypothesized that high infection intensities indicating high loads of virus genomes within individual hosts might promote maintenance due to de novo mutation and adaptation to novel hosts. However, infection intensity was not significantly associated with maintenance after spillover in the single or multiple regression analyses of our correlative approach, and we did not observe experimental lines with highly infected individuals being more likely to emerge (e.g., VX80, JU1428). Infection intensity was incorporated into the mechanistic model through the assumption that it is correlated with the infectiousness of a host, and in our model comparison approach, the Akaike weight of all models that included an additional effect of infection intensity was high, indicating that infection intensity might be an important predictor of passage success beyond its role in our mechanistic model. However, despite this high Akaike weight, the model averaged parameter estimate overlapped zero, calling into question whether infection intensity is truly useful for predicting maintenance. High infection intensity reflects an enhanced ability to replicate virus genomes within host cells, but it is quite possible that this replication is disconnected from other critical steps in the virus life cycle such as virus packaging and release from host cells. Importantly, worm strains VX80 and JU1428 had relatively high infection intensities, but shedding ability and infection prevalence were relatively low, potentially pointing to such a phenomenon. It is possible that a different measure of infection intensity would have yielded clearer patterns, especially since RT-qPCR data on individual worms can be noisy and because expression of RNA1 varies over the course of an infection [44].

Our correlative approach also highlighted the importance of the virus’s ability to exit as an infectious particle from spillover hosts. Shedding was an important predictor of virus maintenance as an individual predictor as well as in the model that included all the epidemiological characteristics. Notably, an additional effect of shedding was not an important predictor in our mechanistic modeling approach, indicating that the role of shedding was reasonably well captured by the mechanistic effect alone. Only one experimental line displayed maintenance for 10 passages without exhibiting noticeable shedding in the spillover population (*C. wallacei* JU1873). Presumably, the sample size for our shedding assay was too small to catch any shedding that occurred in this species, or the virus gained the ability to shed from hosts during early passages of this line. Future studies determining whether and how often an increase in shedding ability occurs in *C. wallacei* (and other non-shedding strains) would be worthwhile, as would elucidating the molecular and ecological mechanisms underlying a gain in shedding ability if it did indeed occur.

The relative susceptibility of the strains correlated with maintenance in our single-factor model but not when other epidemiological characteristics were included. These mixed results likely arose due to its correlation (correlation coefficient ρ = 0.44) with infection prevalence (S4 Fig). Thus, if relative susceptibility were possible to assess or predict in natural populations, it could potentially be used as an indicator of emergence risk in populations likely to experience spillover. One situation where relative susceptibility might be particularly useful is in assessing emergence risk in domesticated crops or livestock where determining relative susceptibility to a pathogen may be feasible through experimental infections [45,46]. Notably, the mechanistic model presumably captured the role of relative susceptibility in virus maintenance since relative susceptibility was not an important predictor of passage success in our model-comparison analysis.

We were surprised to note variation in the characteristics of different strain thaw lines over the course of this experiment as well as differences in patterns of ongoing transmission between this study and our original work in this system [23]. It is possible that the recently isolated wild strains obtained from the *Caenorhabditis* Genetics Center and Marie-Anne Félix used here lose genetic variation during routine passaging in the lab and when coming out of frozen stocks. Loss of genetic variation and founder effects in different lines of the same strain could potentially explain differences in susceptibility and transmission in different experiments, but these differences might also be explained by other unknown block effects which are common in ecological experiments [47].

Existing observational studies have focused on viral or host determinants of spillover and emergence probability. Here, we have shown using a model system that early aspects of spillover ecology, such as the prevalence of infection, the ability of novel hosts to shed the virus, and the relative susceptibility of spillover hosts can be associated with virus maintenance after spillover. These results suggest that situations leading to high infection prevalence of a virus after spillover and those with minimal barriers to shedding should be targeted as high-risk pathogens for emergence. Our results also demonstrate that there are multiple reasons that emergence after spillover might not be achieved since low prevalence, shedding, or relative susceptibility could each individually prevent it. By combining ecological characteristics of spillover events with our existing understanding of viruses that are likely to spillover and emerge, we can improve our ability to identify and halt novel disease emergence.

## Methods

### Worm strains

We used worm strains previously found to be susceptible to Orsay virus with variable maintenance of virus during passaging [23]. These worm strains were *C. sulstoni* strain SB454, *C. latens* strains VX80 and JU724, *Caenorhabditis macrosperma* strain JU1857, *Caenorhabditis tropicalis* strain JU1428, *C. wallacei* JU1873, *C. sp. 25* strain ZF1092, and *C. sp. 65* strain JU4093. Highly susceptible *C. elegans* strain JU1580 [24] was used for positive and negative controls. Although we use the term “strain,” most nematode strains used in this study were isolated from the wild as iso-female or iso-hermaphroditic lines and frozen within a few generations of isolation. Therefore, these strains may contain more genetic variation than typical *C. elegans* laboratory strains that have been inbred through self-fertilization for dozens of generations.

Data were collected across four blocks. Separate from the experiment, strains were maintained at 20 °C and periodically passaged to fresh media and food. Each strain was restarted twice during the experiment from frozen laboratory stocks or from stocks sent from the *Caenorhabditis* Genetics Center, which was the original source of the strains (except for JU4093, which was a gift from Marie-Anne Félix). The two lines of JU724 exhibited markedly different infection dynamics, likely due to host evolution during standard laboratory passaging and/or different genotypes emerging from frozen stocks. We account for this by treating these lines as separate strains (i.e., JU724.1 (blocks 1 and 2) and JU724.2 (block 3)) in statistical models; unfortunately, line JU724.2 was prone to contamination and more difficult to maintain, so our data for JU724 includes 4/4 experimental replicates from each of blocks 1 and 2 (JU724.1), 2/4 experimental replicates from block 3 (JU724.2) and 0/4 from block 4 (JU724.2). The passage history of lines used in the experiment is documented in S1 Table.

### Exposures and passaging

In each block, we initiated four replicate passage lines per strain with five adult mated female worms or five adult hermaphrodite worms exposed to 3 μL virus stock (concentration: 187 × TCID50 of JU1580/ μL, 95% CI: 87.7–360.8 × TCID50 of JU1580/ μL) pipetted to the middle of a lawn of OP50 (grown from 200 μL OP50 in Luria-Bertani (LB) media [48]) on Nematode Growth Medium (NGM; [48]) in 60 mm petri plates. This is the standard exposure method in our laboratory. Two control lines in each block were initiated with five adult *C. elegans* strain JU1580, which is highly susceptible to Orsay virus [24]. One of these lines was exposed to 3 μL Orsay virus stock as a positive control, and one was exposed to 3 μL water as a negative control. Worms were allowed to reproduce and exposed populations grew until they depleted their food (5–13 days). This method ensured that all virus had been consumed before passaging and sampling the worms. Next, 20 adult worms (mated females or hermaphrodites) were passaged to fresh plates seeded with OP50. The remainder of the worms were washed from plates with 1,800 μL M9 buffer [48] into 1.7 mL microcentrifuge tubes. Worms were pelleted by centrifugation at 1,000*g* for 1 min, and supernatants were removed down to 100 μL (including the worm pellet). Worms were washed three times to remove residual virus by adding 900 μL M9, centrifuging at 1,000*g* to re-pellet worms, and removing supernatants down to 100 μL. The washed worms in 100 μL of M9 were then transferred onto NGM plates, and the liquid was allowed to dry over several minutes. Individual or groups of adult worms were then picked for shedding, prevalence, and infection intensity assays (see below). The remainder of the worms were then washed off plates again with 500 μL water into 2 mL snap cap tubes with approximately 100 μL 0.5 mm silica beads and lysed by shaking in a TissueLyser II (Qiagen) for 2 min at a frequency of 30 shakes per second. Lysates were purified through two centrifugation steps at 17,000*g* for 5 min each to separate beads and worm debris from supernatants. The supernatant from the second centrifuge step was saved to test for the presence of Orsay virus RNA1 (which encodes the RNA-dependent RNA polymerase) by reverse transcription quantitative PCR (RT-qPCR) as has been described previously [23].

This extraction process was modified slightly for passaged worm populations to monitor for continued virus transmission. When the populations on passage plates were recently starved, 20 worms were again passaged to new OP50-seeded NGM plates, and worm populations were subjected to the same washing procedure as above except all washing steps were completed with water, and worms were lysed in 500 μL water directly after the three washing steps. Passages continued until virus was no longer detectable in the previous passage plate by RT-qPCR or until the 10th passage plate was processed.

### Determining prevalence and infection intensity

Individual and groups of adult worms (mated females or hermaphrodites) were picked from washed, exposed populations (see above) and placed in 20 μL of 10% proteinase K in 0.2 mL strip tubes. When there were enough adult worms, 16 tubes per population were used: 3 tubes containing individual worms, 3 tubes containing 2 worms, 9 tubes containing 3 worms, and 1 tube without worms but where the worm pick was scraped across the plate to mock picking up a worm and swirled in the proteinase K solution (this was a negative control to ensure that virus was not detected solely due to contamination from the surface of the plate). When there were not enough worms, fewer samples were collected, prioritizing collection of the individual worms and then the two-worm groups. Out of 118 plates processed, we had enough worms for the full collection for 84 plates (72%). Out of all samples, an average of 13.9 tubes with worms (minimum 5, max 15) were used to assess prevalence. Tubes were subjected to a heat treatment in the thermocycler of 60 min at 56 °C followed by 10 min at 95 °C to break down worm tissues and release virus from cells. Samples were preserved at −80 °C until virus RNA could be quantified by RT-qPCR [23]. An individual or group was considered infected if more virus was detected within it than in the negative control plate scrape sample. Virus was only detected in the negative control plate scrape four times across the entire experiment (115 plate scrape samples) with a minimum Ct of 32.3. The maximum likelihood prevalence of infection was calculated from the detection data in the single-, 2-worm, and 3-worm tubes and from the shedding capability assay (below). Median infection intensity was calculated using Ct values from infected single-, 2-worm, and 3-worm samples if there was a greater than 70% chance the groups contained no more than one infected worm (as determined from maximum likelihood prevalence estimates). When groups had more than a 30% chance of containing more than one infected worm, the Ct values from these samples were removed from the median Ct calculation to prevent biasing infection intensities toward more intense infections on plates that had higher prevalence (S5 Fig).

### Determining shedding capability

Three groups of 15 washed adult worms (mated females or hermaphrodites) were picked from washed, exposed populations (see above), and placed onto NGM plates seeded with 200 μL OP50. Five L4-adult worms of the highly susceptible fluorescent pals-5p::GFP *C. elegans* strain *jyIs8;rde-1* [28] were subsequently added to each plate as indicators of shedding. These plates were observed under a fluorescent dissecting microscope for 5 days during which time populations of exposed worms and of the indicator worms replicated. Fluorescing *jyIs8;rde-1* worms indicated that novel hosts were capable of shedding virus and that a sufficient number of infected hosts had been added to the plate to transmit infection after 5 days. We, thus, calculated “shedding ability”—the probability that one infected worm could shed enough virus to make a shedding assay plate glow under the assay circumstances—for each passage line. This metric was estimated alongside prevalence by maximizing the likelihood of each metric given the detection of virus by RT-qPCR in groups of worms in strip tubes (see above) and the number of plates glowing in the shedding assay, using the below equation:


$$\begin{array}{c} L(p,\;y\;|\;g,\;m_{1},\;m_{2},\;m_{3},\;G,\;M_{1},M_{2},\;M_{3})=dbinom(g,\;G,\;1-{\left(1-py\right)}^{15}*\prod_{N=1}^{3}dbinom(m_{N},\;M_{N},\;1-{\left(1-p\right)}^{N}). \end{array}$$ (2)

Above, dbinom(a, b, c) is shorthand for the probability distribution function of a binomial distribution with a successes on b attempts assuming probability of success c. g is the number of plates on which glowing was detected, G is the total number of plates on which shedding ability was assayed, p is the estimated prevalence of infection, and y is the estimated probability of shedding given a worm is infected. Note that the number 15 appears in this equation because 15 worms were simultaneously transferred for each shedding assay replicate. In addition, $m_{N}$ is the number of tubes with N worms that tested positive for virus by RT-qPCR, and $M_{N}$ is the total number of tubes with N worms. Using the above equation, values of  p and y were estimated for each exposure plate using a grid search in R where the maximum likelihood of the data was determined from a grid of values of p and y, where p and y each ranged from 0 to 1 by increments of 0.01.

### Determining relative susceptibility

The relative susceptibility of each strain was determined by measuring the median tissue culture infectious dose (TCID50) per μL of our stock of Orsay virus assayed in each strain and comparing these to the TCID50 per μL of the stock virus assayed in highly susceptible control strain *C. elegans* JU1580. We performed TCID50 assays in 4 blocks where each block contained 3–5 experimental strains, *C. elegans* strain JU1580, and no-worm controls. TCID50 assays were conducted using 24-well tissue culture plates where wells were filled with approximately 2 mL of NGM and spotted with 20 µL OP50. OP50 spots were allowed to grow for approximately 24 hours before 20 µL of diluted stock virus was added to the top of spots, covering them entirely. We exposed triplicate populations of 50 L1 stage worms to five virus doses that varied by factors of 10 from 3.33 × experimental exposure dose to 0.00033 × experimental exposure dose. Worms were exposed for 3 days at 20 °C by which time they reached adulthood and eggs were visible in the wells. Worms were then washed out of wells with 1 mL water and pipetted into 1.7 mL microcentrifuge tubes. Worms were condensed into a pellet by centrifugation and washed once by removing 900 µL supernatant and adding 900 µL water. Tubes were centrifuged again to pellet worms before supernatant was removed to 500 µL. Then, worms and water were transferred to 2 mL round bottom snap-cap tubes with silica beads for tissue lysis and centrifugation as above. Samples were preserved at −80 °C until virus quantification by RT-qPCR. Populations were considered infected if more virus was detected than in the no-worm controls for the same dose. The TCID50 per μL for each strain/thaw line was determined by predicting the likelihood of the data (fraction of populations infected at each virus dose) over a wide range of potential TCID50 values and selecting the maximum likelihood value with two exceptions: JU1428-line 1 and VX80-line 2, where assays failed due to contamination (S6 Fig, S2 Table). Except for the two thaw lines of JU724, TCID50 per μL estimates for strains of different thaw lines did not differ significantly from each other by likelihood ratio tests. Thus, maximum likelihood estimates of TCID50 per μL were re-calculated over the data from both thaw lines when available, except for JU724 where TCID50s per μL for different thaw lines were kept separate (see Fig 2E).

### Statistical analyses

Analyses were carried out in R version 4.3.1 [49]. We modeled differences in epidemiological characteristics of spillover (i.e., prevalence, shedding ability, and median infection intensity) among strains and blocks using (generalized) linear models in R package lme4 version 1.1.31 [30]. Differences in prevalence and shedding ability were modeled using the quasibinomial family, whereas differences in median infection intensity were modeled with the Gaussian family. Differences in relative susceptibility (TCID50/μL of the stock virus measured in each strain/thaw line divided by the TCID50/μL measured in *C. elegans* JU1580) were assessed by likelihood ratio tests (see above).

To determine which epidemiological characteristics are important drivers of virus maintenance after spillover, we modeled the number of successful virus passages with negative binomial mixed-effects models using lme4 version 1.1.31 [30]. Prevalence, shedding ability, infection intensity, and relative susceptibility were modeled as fixed effects in separate models as individual predictors and in one model all together while strain and block were included as independent random effects in all models. We assessed importance of factors in models by likelihood ratio tests and the calculation of marginal R^2^ values [50] using R package performance version 0.10.4 [51].

### Mechanistic model predicting passage success

To better understand the mechanism of maintenance, we constructed a mechanistic model that used the parameters that we inferred or measured, to estimate the probability of passage success, assuming passage occurs when one or more worms are transferred that are capable of initiating infection on a new plate. Our model for successful passage is therefore the probability that an infected worm is selected, multiplied by the chance that the worm was infectious, multiplied by the chance that the worm was infectious enough to infect conspecifics. Parameters in the model are described in Table 3. In practice, to determine the chance that a worm is infectious enough to infect conspecifics, we had to make several assumptions. First, we assumed that infectiousness was related to a worm’s infection intensity (Ct), such that a lower Ct indicated a more infectious worm. Second, we assumed that the relative susceptibility of a strain was directly proportional to the Ct required to cause infection, such that less susceptible strains required a higher infection intensity (lower Ct) to successfully transmit infection. Given these two assumptions, we generated per-plate estimates of the median Ct (μ) modeled using a linear mixed-effects model, where there was a fixed effect of prevalence and a random effect of strain (S7 Fig). Using this model, we used the square root of the residual variance to approximate the standard deviation of Ct (σ) between different infected worms on the same plate. Thus, under this set of assumptions, the probability that a particular worm will be infected, shedding, and have a high enough intensity of infection to transfer infection to a new plate is $p_{i}y_{i}z_{ij}$, where $p_{i}$ is again the prevalence of infection on replicate plate i, $y_{i}$ is the fraction of infected worms on replicate plate i that are shedding, and $z_{ij}$ is the fraction of infected worms on replicate plate i shedding enough to infect strain j. In practice, $z_{ij}$ was modeled using the cumulative distribution of the normal distribution such that $z_{ij}=Prob(x<40+\log_{2}s_{j})$, where x is normally distributed with mean $\mu_{i}$ and standard deviation σ, and $s_{j}$ is the relative susceptibility of the strain. Thus, the overall probability of passage success for a particular replicate plate i and strain j can be written as 1 minus the probability of passage failure, or equivalently:

**Table 3:** Explanation of parameters in mechanistic model.

Parameter	Explanation
$p_{i}$	Infection prevalence on spillover plate i
$y_{i}$	Probability that a worm from plate i is shedding given that it is infected
$z_{ij}$	Probability that an infected worm from plate i is shedding enough to transfer infection to a plate of strain j
$\mu_{i}$	Estimated median intensity of infection (measured on Ct scale) for infected worms on plate i
σ	Estimated standard deviation of infection intensity (measured on Ct scale)
$s_{j}$	Relative susceptibility of strain j (TCID50/μL of the stock virus measured in strain j divided by the TCID50/μL measured in *C. elegans* JU1580)


$$Probability\;of\;passage=\;1-{\left(1-p_{i}y_{i}z_{ij}\right)}^{20}$$ (3)

We then adjusted our mechanistic expectations by weighting the calculations from the mechanistic model by 0.8 and adding the remaining weight (0.2) times a 50% chance of passage. This adjustment moves our estimates away from extremes and toward intermediate probabilities of passage success, thereby avoiding 100% and 0% chances of passage and accounting for uncertainty in our estimates.

We analyzed the quality of our mechanistic model by regressing the actual passage results against the logit transform of the weighted mechanistic model results with a binomial generalized linear model and with a binomial generalized linear mixed-effects model where strain, experimental line, passage number, and block were included as random effects. The intercept was set to 0 in both models since we expected a 1 to 1 relationship between actual and predicted passage success. We considered a passage successful if Ct < 25 for these and following analyses, since in our prior work we have found that passage success is extremely unlikely when the Ct of the whole plate extraction is above 25. We calculated the R^2^ of the generalized linear model using R^2^ = 1 − (deviance/null deviance), and we calculated the marginal R^2^ of factors in the mixed models using marginal R^2^ = variance of a given effect (i.e., fixed or random)/(sum of the variances of each effect + π^2^/3) [50] or with the R package MuMIn version 1.48.4 [31].

We then used the logit transform of the weighted mechanistic model results as an offset in a suite of 16 binomial generalized linear mixed-effect models that included or excluded each of the epidemiological characteristics as fixed effects to determine which if any were important beyond their role in our mechanistic model. The random effects of strain, experimental line, passage number, and block were included in all models as was the offset. We used AICc to determine the best model and AICc weights to make relative comparisons between models. The AICc weight of a given model k is calculated as $\begin{array}{c} e^{-\Delta AICc_{k}/2}/\sum_{j}e^{-\Delta AICc_{j}/2} \end{array}$ where ΔAICc is the difference between the AICc of a given model and the best model, and j is the set of all models [52]. We also calculated AICc weights of model components to determine the importance of a given epidemiological characteristic in models to predict passage success [52]. The AICc weight of a model component is calculated as $\begin{array}{c} \sum_{i}e^{-\Delta AICc_{i}/2}/\sum_{j}e^{-\Delta AICc_{j}/2} \end{array}$ where i refers to the set of models that includes a given parameter and j is the set of all models, and ΔAICc is the difference between the AICc of a given model and the best model [52]. Finally, we calculated model-averaged parameter estimates using package AICcmodavg version 2.3.2 [53]. All figures were produced with package ggplot2 version 3.4.0 [54].

## Supporting information

S1 Fig Associations between maximum likelihood prevalence estimates and passages until virus loss by strain. The data underlying this figure can be found in https://doi.org/10.5281/zenodo.15739577.
(TIFF)

S2 Fig Associations between average infection intensity (median Ct value) and passages until virus loss by strain. The data underlying this figure can be found in https://doi.org/10.5281/zenodo.15739577.
(TIFF)

S3 Fig Associations between maximum likelihood shedding ability estimates and passages until virus loss by strain. The data underlying this figure can be found in https://doi.org/10.5281/zenodo.15739577.
(TIFF)

S4 Fig Correlations between epidemiological characteristics of spillover for individual passage lines. Correlation coefficients are noted in red. The data underlying this figure can be found in https://doi.org/10.5281/zenodo.15739577.
(TIFF)

S5 Fig We corrected infection intensity (median Ct) by using only data from extractions with a greater than 70% chance they contained no more than one infected worm (as determined from maximum likelihood prevalence estimates). A) If intensities were corrected, they were mostly corrected to have lower infection intensities. B) This reduces bias toward higher median infection intensities at higher infection prevalence. The filled and fully back circles represent points that did not have to be corrected (greater than 70% chance they contained no more than one infected worm); the open circles with black outlines represent uncorrected median Ct values that needed to be corrected; the filled black circles enclosed with red represent corrected median Ct values. The data underlying this figure can be found in https://doi.org/10.5281/zenodo.15739577.
(TIFF)

S6 Fig TCID50 determinations for each strain and thaw line of TCID50 assays with 95% confidence intervals. Data are color coded by strain. The data underlying this figure can be found in https://doi.org/10.5281/zenodo.15739577.
(TIFF)

S7 Fig Predicted median Ct correlates with the corrected median Ct values. The predicted values were modeled using a linear mixed-effects model, where there was a fixed effect of prevalence and a random effect of strain. The data underlying this figure can be found in https://doi.org/10.5281/zenodo.15739577.
(TIFF)

S1 Table Passage history of strains used across the four blocks of the experiment. Each strain was restarted twice during the experiment from frozen laboratory stocks or from stocks sent from the *Caenorhabditis* Genetics Center, which was the original source of the strains (except for JU4093, which was a gift from Marie-Anne Félix).
(XLSX)

S2 Table TCID50 determinations for each strain and thaw line. (XLSX)

## Abbreviations

LB: Luria-Bertani
NGM: nematode growth medium
