# Supplementary figures and images for: Early epidemiological characteristics explain the chance of population-level virus persistence following spillover events
Source: PLoS Biol. 2025 Aug 21;23(8):e3003315. doi: 10.1371/journal.pbio.3003315 (PMC12370034; doi:10.1371/journal.pbio.3003315)

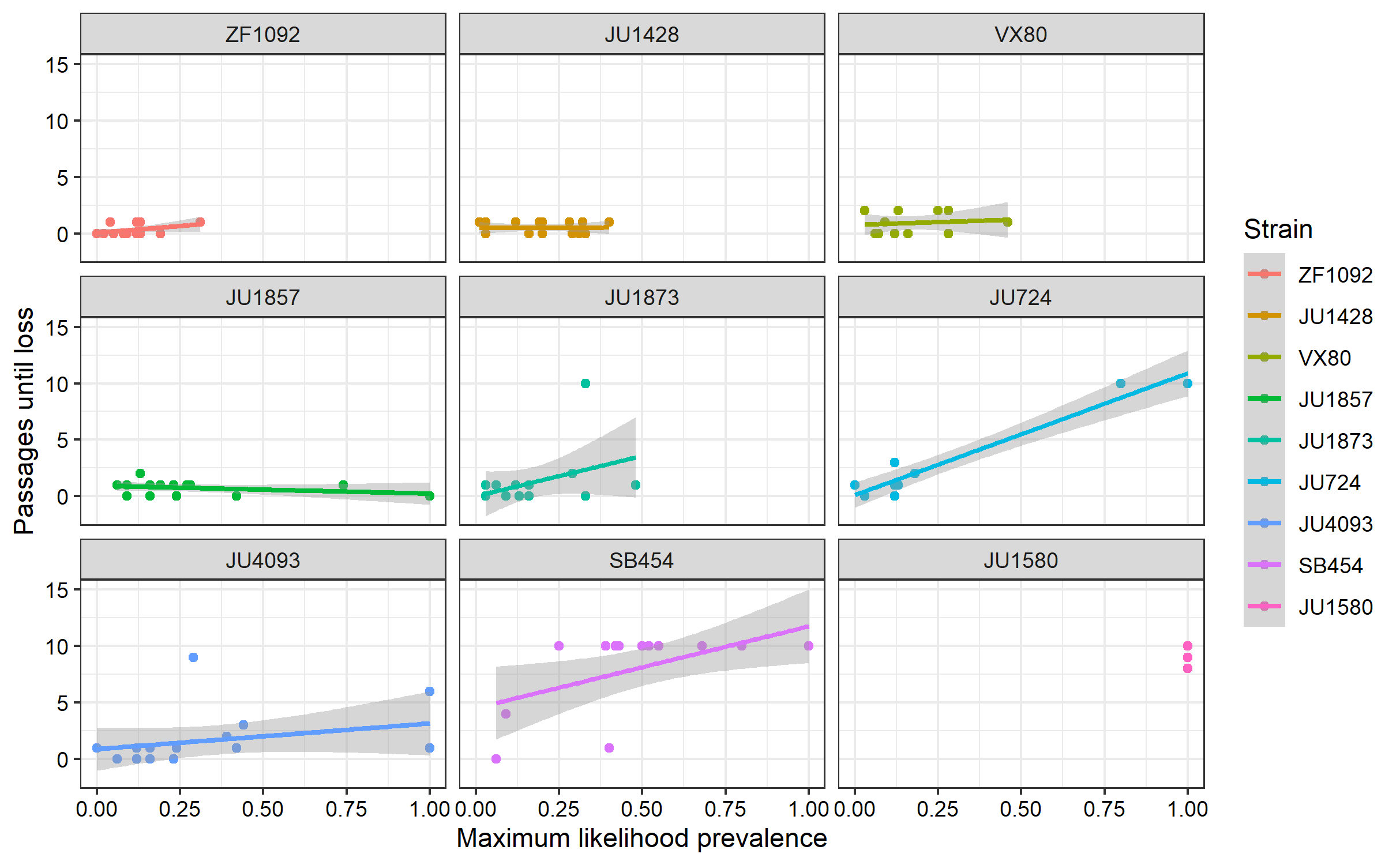

Supplement: S1 Fig — The data underlying this figure can be found in https://doi.org/10.5281/zenodo.15739577. (TIFF) [file pbio.3003315.s001.tiff]

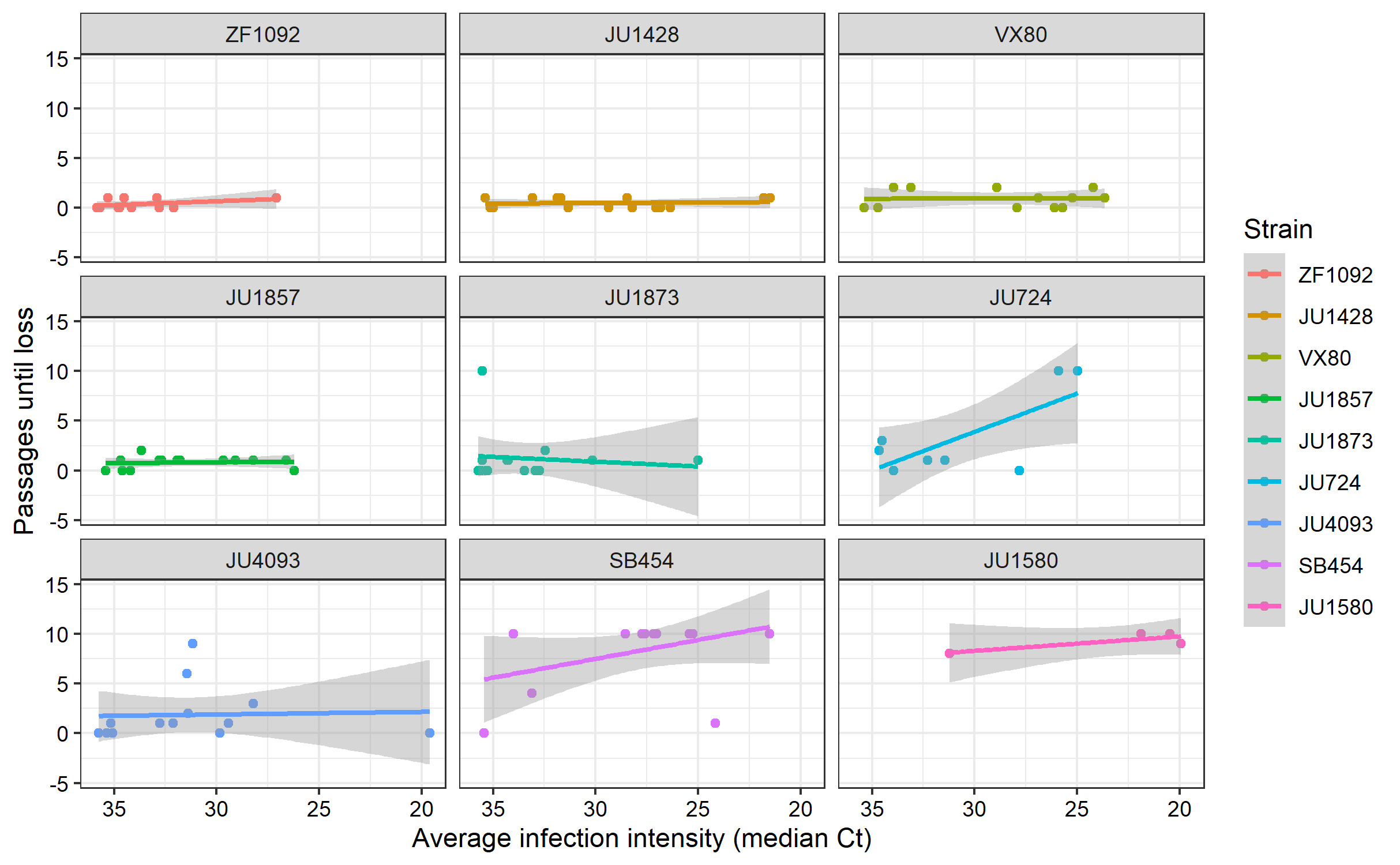

Supplement: S2 Fig — The data underlying this figure can be found in https://doi.org/10.5281/zenodo.15739577. (TIFF) [file pbio.3003315.s002.tiff]

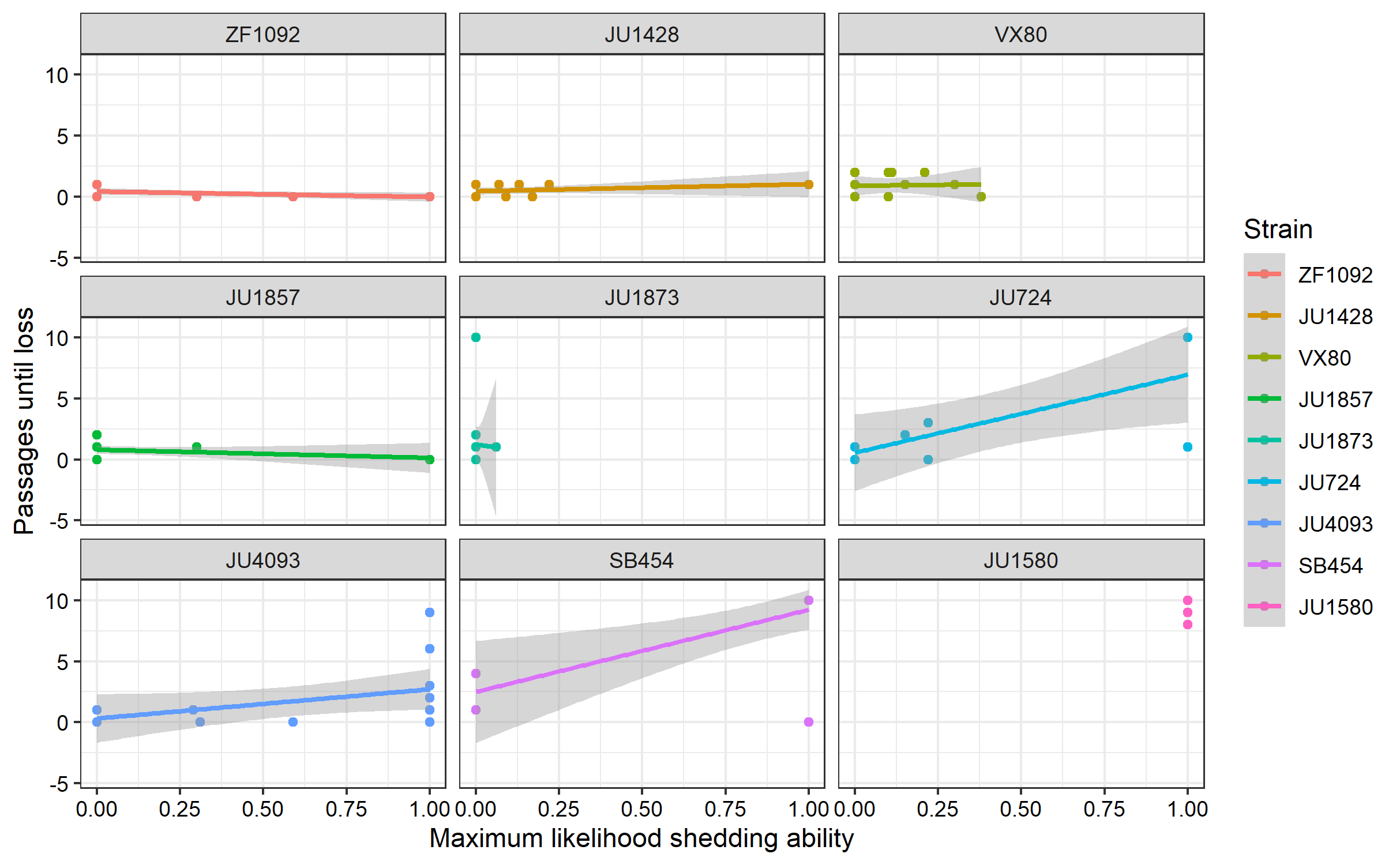

Supplement: S3 Fig — The data underlying this figure can be found in https://doi.org/10.5281/zenodo.15739577. (TIFF) [file pbio.3003315.s003.tiff]

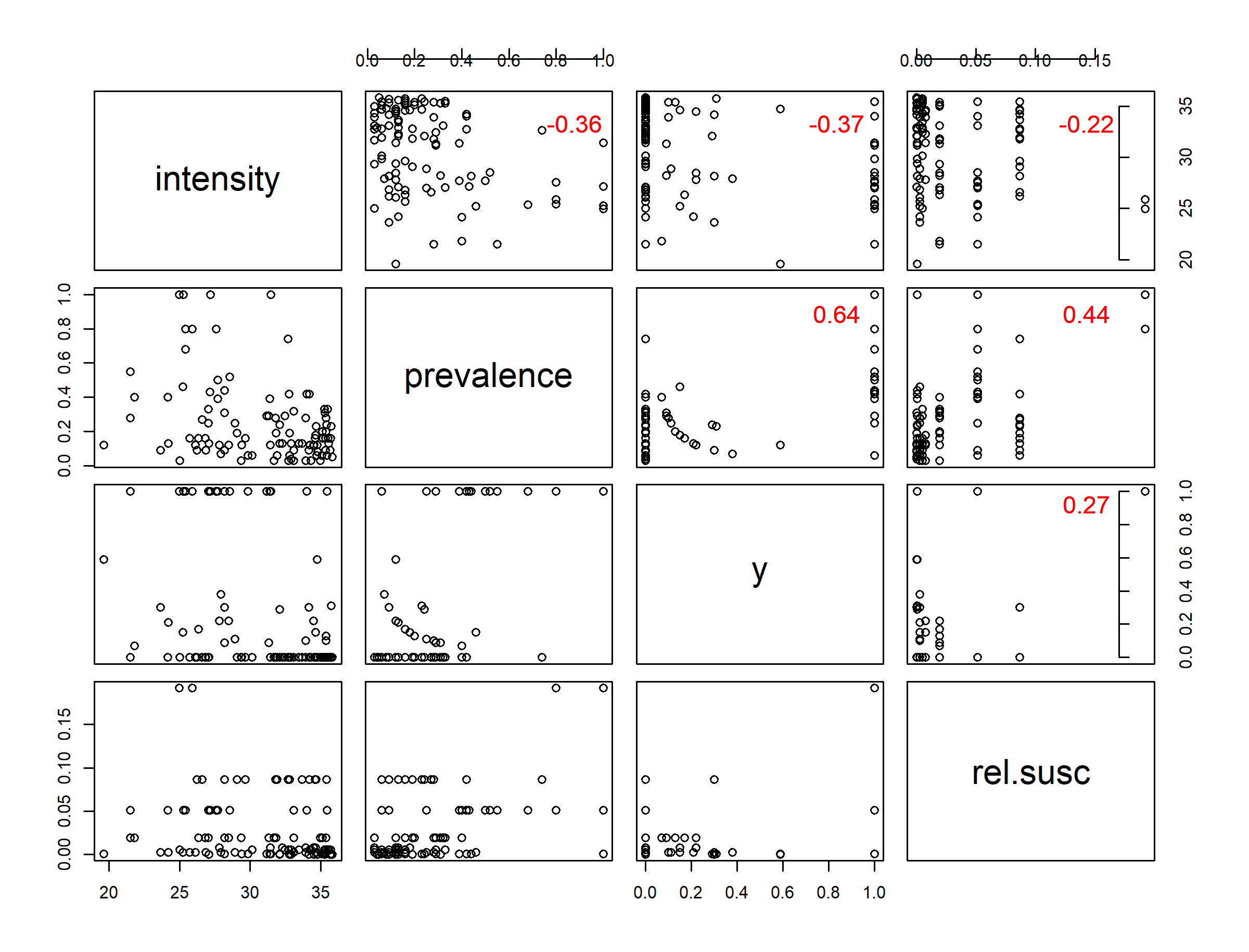

Supplement: S4 Fig — Correlation coefficients are noted in red. The data underlying this figure can be found in https://doi.org/10.5281/zenodo.15739577. (TIFF) [file pbio.3003315.s004.tiff]

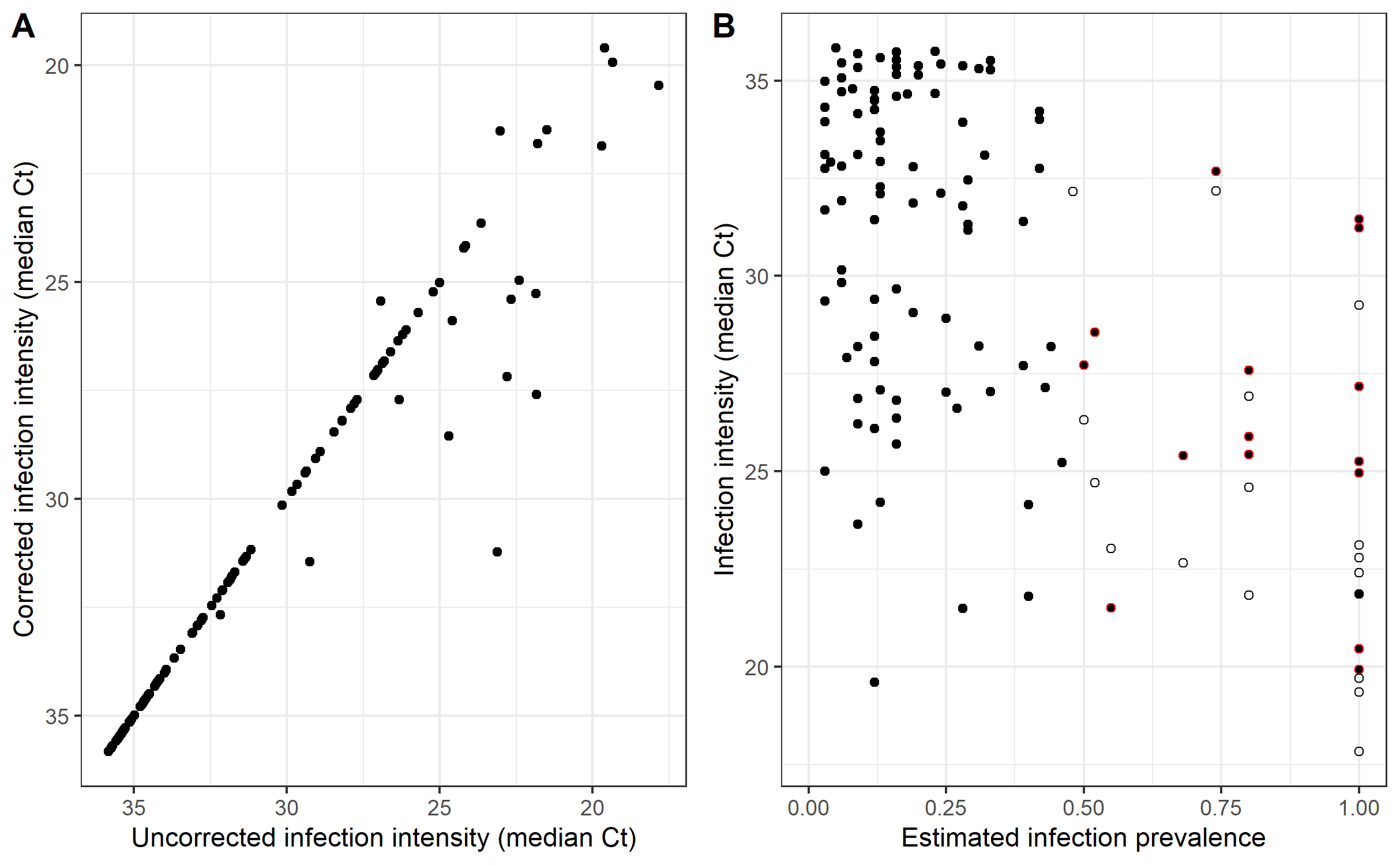

Supplement: S5 Fig — A) If intensities were corrected, they were mostly corrected to have lower infection intensities. B) This reduces bias toward higher median infection intensities at higher infection prevalence. The filled and fully back circles represent points that did not have to be corrected (greater than 70% chance they contained no more than one infected worm); the open circles with black outlines represent uncorrected median Ct values that needed to be corrected; the filled black circles enclosed with red represent corrected median Ct values. The data underlying this figure can be found in https://doi.org/10.5281/zenodo.15739577. (TIFF) [file pbio.3003315.s005.tiff]

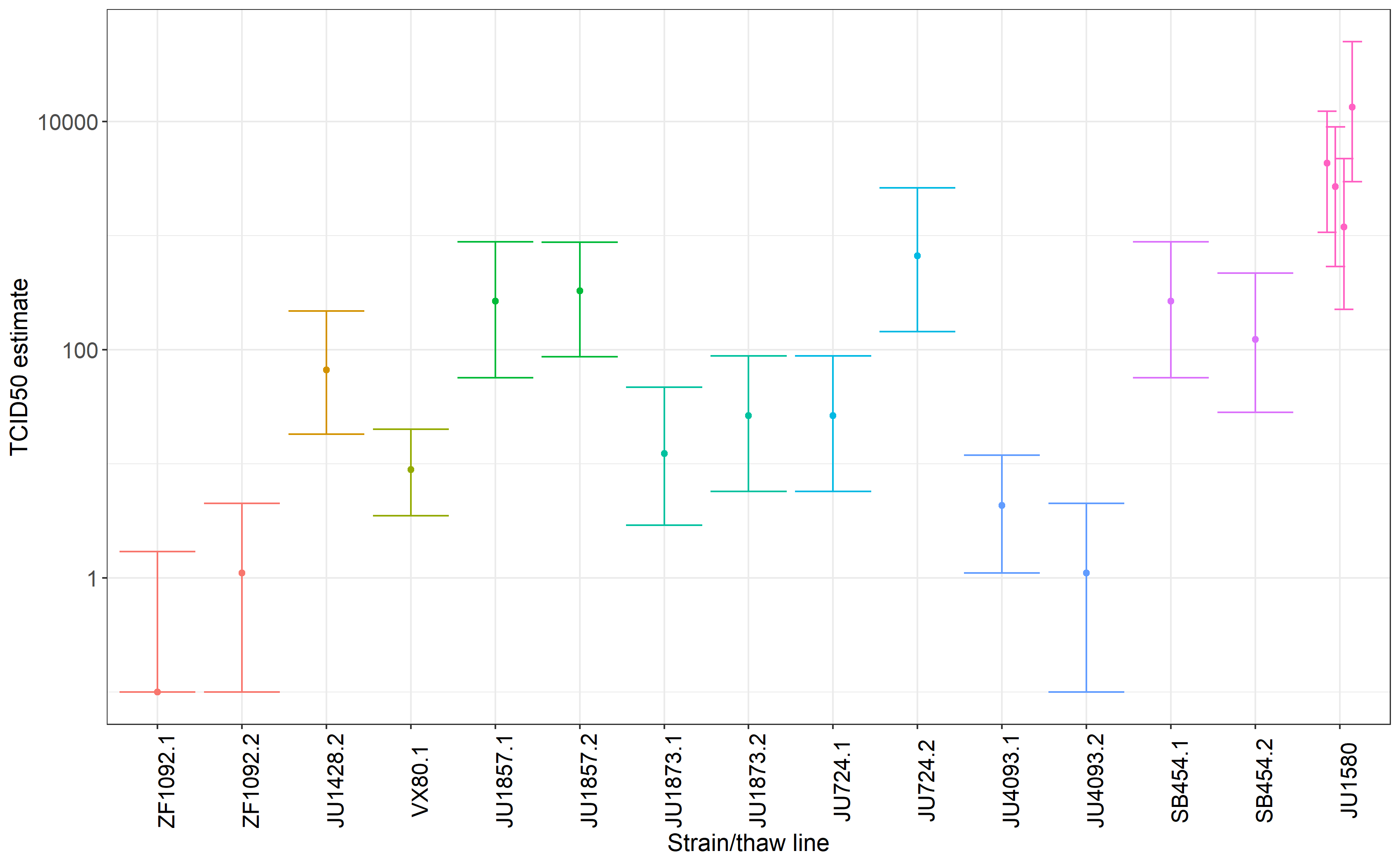

Supplement: S6 Fig — Data are color coded by strain. The data underlying this figure can be found in https://doi.org/10.5281/zenodo.15739577. (TIFF) [file pbio.3003315.s006.tiff]

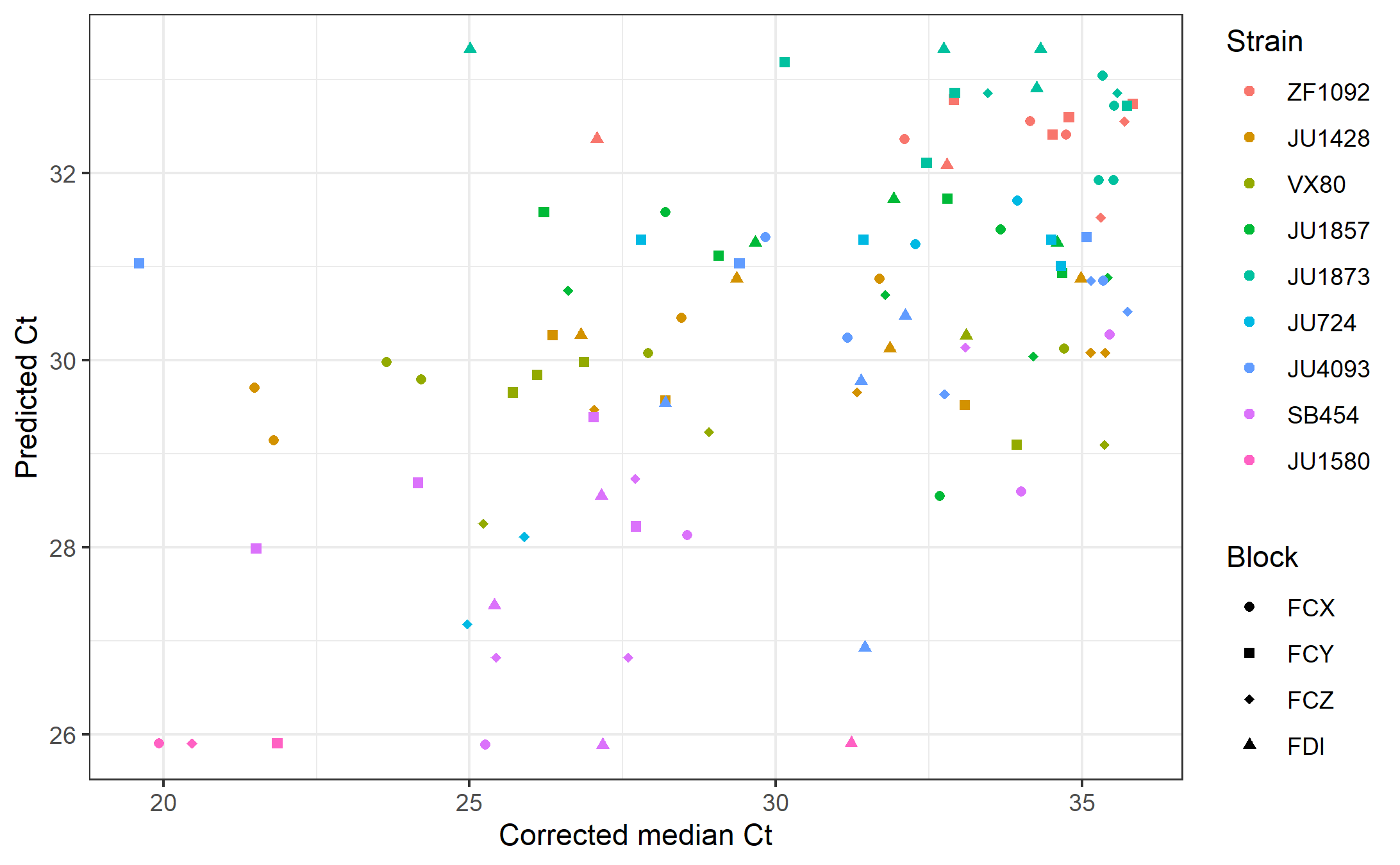

Supplement: S7 Fig — The predicted values were modeled using a linear mixed-effects model, where there was a fixed effect of prevalence and a random effect of strain. The data underlying this figure can be found in https://doi.org/10.5281/zenodo.15739577. (TIFF) [file pbio.3003315.s007.tiff]
